# An extended multiplicative error model of allometry: Incorporating systematic components, non-normal distributions, and piecewise heteroscedasticity

**DOI:** 10.1093/biomethods/bpae024

**Published:** 2024-04-18

**Authors:** Héctor Echavarría-Heras, Enrique Villa-Diharce, Abelardo Montesinos-López, Cecilia Leal-Ramírez

**Affiliations:** Centro de Investigación Científica y de Estudios Superiores de Ensenada, Carretera Ensenada-Tijuana No. 3918, Zona Playitas, Ensenada, B.C., México; Centro de Investigación en Matemáticas, A.C. Jalisco s/n, Mineral Valenciana, Guanajuato Gto., 36240, México; Departamento de Matemáticas, Centro Universitario de Ciencias Exactas e Ingenierías (CUCEI), Universidad de Guadalajara, 44430, Guadalajara, Jalisco, México; Centro de Investigación Científica y de Estudios Superiores de Ensenada, Carretera Ensenada-Tijuana No. 3918, Zona Playitas, Ensenada, B.C., México

**Keywords:** comprehensive multiplicative error model, allometry, biphasic systematic term, normal mixture distribution, piecewise-like heteroscedasticity

## Abstract

Allometry refers to the relationship between the size of a trait and that of the whole body of an organism. Pioneering observations by Otto Snell and further elucidation by D’Arcy Thompson set the stage for its integration into Huxley’s explanation of constant relative growth that epitomizes through the formula of simple allometry. The traditional method to identify such a model conforms to a regression protocol fitted in the direct scales of data. It involves Huxley’s formula-systematic part and a lognormally distributed multiplicative error term. In many instances of allometric examination, the predictive strength of this paradigm is unsuitable. Established approaches to improve fit enhance the complexity of the systematic relationship while keeping the go-along normality-borne error. These extensions followed Huxley’s idea that considering a biphasic allometric pattern could be necessary. However, for present data composing 10 410 pairs of measurements of individual eelgrass leaf dry weight and area, a fit relying on a biphasic systematic term and multiplicative lognormal errors barely improved correspondence measure values while maintaining a heavy tails problem. Moreover, the biphasic form and multiplicative-lognormal-mixture errors did not provide complete fit dependability either. However, updating the outline of such an error term to allow heteroscedasticity to occur in a piecewise-like mode finally produced overall fit consistency. Our results demonstrate that when attempting to achieve fit quality improvement in a Huxley’s model-based multiplicative error scheme, allowing for a complex allometry form for the systematic part, a non-normal distribution-driven error term and a composite of uneven patterns to describe the heteroscedastic outline could be essential.

## Introduction

Allometry, also known as biological scaling, refers to the relationship between the size of a trait and that of the whole body of an organism. Pioneering observations by Otto Snell in 1892 and further elucidation by D’Arcy Thompson [1] set the stage for its integration into Huxley’s explanation of constant relative growth [1–4]. It epitomizes through Huxley’s formula of simple allometry expressing through the power function.

(1) $$y=\beta x^{\alpha }$$

The response y and covariate x stand for measurable traits, and α and β nominate as allometric exponent and normalization constant one to one. Equation (1) entails a great practical convenience in circumstances when direct response’s measurements are problematic by offering proxy values obtained using estimates for α and β, and measured values of the covariate. Hence, allometric methods are extensively used in research problems in varied disciplines (e.g. biology [5], biomedical sciences [6], earth sciences [7], resource management [8], and economics [9]).

The traditional method to identify Huxley’s formula conforms to a regression protocol fitted in the direct scales of data. It involves a systematic part given by Equation (1) and a lognormally distributed error term that enters multiplicatively. In what follows, we will refer to such a scheme as a Multiplicative Error Model (MEM). A MEM protocol formally expresses through,

(2) $$y=\beta x^{\alpha }\psi \left(\epsilon \right),$$

where

$$\psi \left(\epsilon \right)=e^{\epsilon }$$

being ϵ a normally distributed random variable with zero mean and standard deviation σ that is, ϵ∼N(0,σ).

The analysis could be carried away through an equivalent protocol fitted in geometrical scales. This bears a simple linear regression model deriving from log-transformation procedures applied to both sides of Huxley’s power function model (e.g. [10–11]). Such a scheme is referred to here as a log-transformation method (LM). The associating LM arrangement becomes,

(3) $$v=\beta_{0}\;+\;\alpha u\;+\;\epsilon$$

where v=ln(y), u=ln(x) and $\beta_{0}=\ln(\beta ).$

Views sustain that a log-transformation approach entails biased results (e.g. [12–20]), thereby endorsing that analysis must keep in direct scales and rely on the MEM scheme or else using a Direct Nonlinear Regression method (DNLR in what follows) [21–25]. Defenders of this perspective claim that inherent stochasticity better fits into an Additive Error Model (AEM subsequently). Nevertheless, the DNLR approach could also face caveats [26, 27]. The AEM formally expresses through the equation:

(4) $$y=\beta x^{\alpha }\;+\;\psi (\epsilon )$$

where ψ(ϵ)= ϵ with ϵ as described around Equation (1). In the settings of the AEM scheme of Equation (4), we often consider a Breusch–Pagan [28] modification. Resulting scheme recognizes here as an AEM-BP model and allows consideration of heteroscedasticity of the allometric response. Formally, a DNLR-BP adaptation assumes ϵ being normally distributed random variable, having a zero mean, but instead of bearing a constant deviation σ, we assume a covariate dependent deviation σ(y|x) that is, $\epsilon ∼N\left(0,\sigma (y|x)\;\right)$. To offer a suitable candidate form, we recall the procedure yielding the Breusch–Pagan [28] test. Consequently, the identification of the AEM or the AEM-BP alternates should be performed in arithmetic scales.

A characterization relating to the AEM provides a suitable protocol for the examination of non-through the origin allometric patterns [19]. Sartori and Ball [16] challenged the traditional understanding of Thracia’s ligamental apparatus, highlighting the existence of distinct layers of fibrous ligament and providing insights into their developmental stages and functional roles. The application of allometric models by Sartori and Ball [16] added quantitative rigor to the analysis. They relied on two- and three-parameter power functions (simple and full allometric equations) to analyze measurements of different ligament parts. Notably, Sartori and Ball [16] recommended to use the full allometric model since the external (parivincular) ligament emerges at a shell length of approximately 2.5 mm, that is the analysis should rely on a non-through the origin allometric protocol.

In many instances of allometric examination, the predictive strength of Huxley’s formula bears unsuitable. Therefore, one linked relevant research problem concerns the accuracy of projections of the response deriving from the MEM, LM, or AEM protocols. Established approaches to improve fit of these schemes enhance the systematic relationship's complexity while keeping the go-along normality-borne errors assumption. These extensions followed Huxley’s own idea that the consideration of a biphasic allometric pattern could render necessary [4]. However, examination of present data reveals that relying on a biphasic form for the systematic term barely improved correspondence measure values though still maintaining a heavy tails problem. Moreover, said biphasic form and errors conforming to a normal-mixture distribution did not provide complete fit consistency either. However, updating the outline of the error term to allow heteroscedasticity to occur in a piecewise-like mode finally produced overall fit consistency. Our results demonstrate that when attempting to achieve fit quality improvement in the MEM scheme, allowing for a complex allometry form for the systematic part, a non-normal distribution-driven error term and a composite of uneven patterns to describe the heteroscedastic outline could be essential. This work devotes at explaining the involved procedures.

## Materials and methods

For present aims, the MEM scheme of Equation (2) generalizes into.

(5) $$y=w\left(x,p\right)\;\psi \left(\;x,\epsilon \right).$$

Involving a continuous function $w\left(x,p\right),\;$of the covariate with $p=(p_{1},\ldots ,\;p_{n})$ a parameter set, and

(6) $$\psi \left(x,\epsilon \right)\;=\;\exp(h\left(x,c\right)\epsilon )$$

involving a variance scaling function h(x,c) where c stands for a parameter set. The random variable ϵ assumes to be ϕ- distributed with zero mean and standard deviation σ, that is $\epsilon ∼\phi \left(0,\sigma \right).$ The protocol entailed by Equation (5) will refer to as the Extended Multiplicative Error Model (EMEM). In what follows, the characterization of the EMEM composing a mean response function $w\left(x,p\right),$ a variance scaling function h(x,c), and a distribution ϕ(0,σ) symbolizes by means of a $(\mathrm{EMEM},w\left(x,p\right)$, h(x,c), ϕ) 4-tuple. The symbols $\mu EM\left[w,h,\phi \right]\left(y|x\right)$ and ${\sigma EM\left[w,h,\phi \right]}^{2}\;\left(y|x\right)$ will stand for the EMEM mean and variance of the response y at covariate value x that are brought by the $(\mathrm{EMEM},w\left(x,p\right)$, h(x,c), ϕ) arrangement. Correspondently, an Extended LM (ELM) expands the complexity of the model of Equation (3) and allows parallel examination of the EMEM in geometrical space. It derives by using a log-transformation u=lnx and v=ln(y) on both sides of Equation (5). Through this work, we confine the use of a log-transformation approach only as a graph assessment tool. For the present aims, the LM or ELM are not intended for model identification purposes. The AEM composite described around Equation (4) is further referred to by means of the $\left(\mathrm{AEM}-BP,\;w_{H}\;N\right)$ triplet. Accordingly, in what follows the three-parameter power function model recommended by Sartori and Ball [16] will be distinguished by a $\left(\mathrm{AEM},\;w_{H}\;+\;c,\;N\right)$ triplet. Recall that in the present examination, we are dealing with an extension of Huxley’s power function-based multiplicative error model that concerns to the analysis of allometric settings that pass through the origin (see Packard [23] and references therein). Therefore, we include the $\left(\mathrm{AEM},\;w_{H}\;+\;c,\;N\right)$ only for comparison of the presently offered model to existing ones.

We assume that $w\left(x,p\right)$ achieves two forms. One becomes Huxley’s power function and denotes by $w_{H}\left(x,p\right),\;$namely,

(7) $$w_{H}\left(x,p\right)=\beta {x^{\alpha }}^{\;}$$

then, $p=\left(\alpha ,\beta \right).$ Alternatively $w\left(x,p\right)$ conveys through Huxley’s biphasic form $w_{BH}\left(x,p\right)$ [4] defined by

(8) $$w_{BH}\left(x,p\right)\;=\beta {{f\left(x\right)}^{\alpha }}^{\;}{g(x)}^{\lambda }$$

where

fx= x if 0 ≤ x ≤ xbxb if xb < x ≤ xmax

gx=  1 if 0 ≤ x ≤ xb x/xb if xb < x ≤ xmax.

In this arrangement p=(β, α, λ, $x_{b})\;$with $x_{b}$ a breakpoint for transition from a first, phase $y=\beta {x^{\alpha }}^{\;}$ holding on $x\;\leq \;x_{b}$ and a second one $y=\beta {x_{b}^{\alpha -\lambda }x^{\lambda }}^{\;}$ working on $x\;>\;x_{b}$. Besides $x_{\max}$, stands for the maximum covariate value.

Correspondingly, ϕ(0,σ) could hold either a normal distribution form, or else that one of a mixture of n normal distributions each one with zero mean and a standard deviation s. We denote such a mixture distribution utilizing the symbol ${NM}_{n}\left(m,\;s\right)$ with m standing for the mean of the mixture and s for the corresponding standard deviation.

## Data

Present data set includes 10 410 pairs of individual eelgrass leaf dry weight y[g] and area x[${\mathrm{mm}}^{2}$] measurements obtained from a sampling carried out over a period of 13 months, in San Quintin Bay Mexico [29]. Supplementary Table S1 describes the distribution pattern, in terms of quantiles, for the 10 412 measurements of eelgrass leaf weights and related areas. Supplementary Table S2 shows the variation pattern of the 7840 observations resulting after applying to raw data a Median Absolute Deviation data quality control procedure [29]. A similarity in the distribution before and after data processing can be perceived in the quantile–quantile (Q–Q) graphs observed in Supplementary Fig. S1. Supplementary Figs S2 and S3 display boxplots for the sampling scheme for raw and processed data one-to-one. From month 2 to month 6, an increase in seawater temperature occurred. This could be behind a corresponding reduction in the values of both leaf dry weight and linked area displayed in raw data. Processed data exhibits similar dynamics. Moreover, the overall variation patterns of raw and processed data throughout the 13 months of sampling are similar. The great similarity of the fitted straight lines for both sets of observations in Supplementary Table S3 confirms that the referred distribution pattern does not change after data processing, even though data processing eliminated a significant number of discrepant observations (23%). Perhaps the referred correspondence could be explained by a lack of standardization of data-gathering routines.

## Results and discussion

### Preliminary graph examination

**Figure 1.**
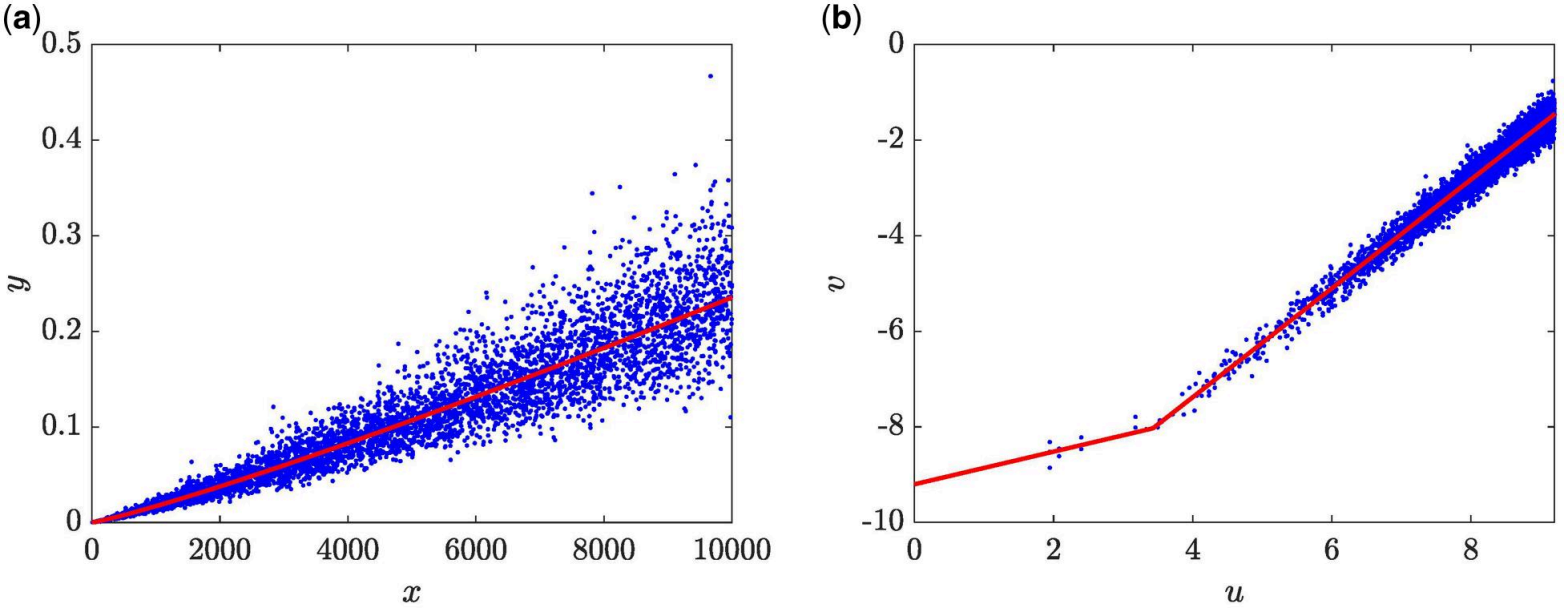
Benefits of the log transformation procedure at unraveling a masked allometric pattern. (a) Biphasic data simulated by Equation (8) shows the dispersion around the $w_{BH}\left(x,p\right)$ curve in arithmetical space. (b) Dispersion around the composition of lines fitted in geometric space to the logarithmically transformed simulated two-phase allometric data.

Packard [20] stresses the relevance of preliminary graph assessment in the allometric examination. Mainly, it is in this respect that a log transformation provides elucidation advantages. In the materials and methods section, we stated that a log transformation approach confines solely to graph assessment. To explain this point, we rely on a simulated data set deriving from a $\left(\mathrm{EMEM},w_{BH},\;1,\;N\right)$ 4-tuple. Accordingly, data points in Fig. 1a were obtained based on a biphasic-multiplicative error model, arrangement, that is, with $w_{BH}\left(x,p\right),$ given by Equation (8), and with $\epsilon ∼N\left(0,\sigma \right).$ Most of the parameter values required to produce the simulated data were taken from Table S6 in the Supplementary file section that is, β=1.008219e-04, α=3.416174e-01, λ=1.138851e + 00, $x_{b}=3.082518e\;+\;01$, but we adapted σ=0.1. Then to produce data points in Fig. 1b, we applied a log transformation. Therefore Fig. 1a displays the resulting simulated biphasic allometric data in the direct scales. We notice that displaying the simulated pairs in arithmetical scales masks an expected distribution given the data-generating procedure. Conversely, Fig. 1b corresponding to $\left(\mathrm{ELM},w_{BH},\;1,\;N\right)$ fit unravels the underlying biphasic pattern.

**Figure 2.**
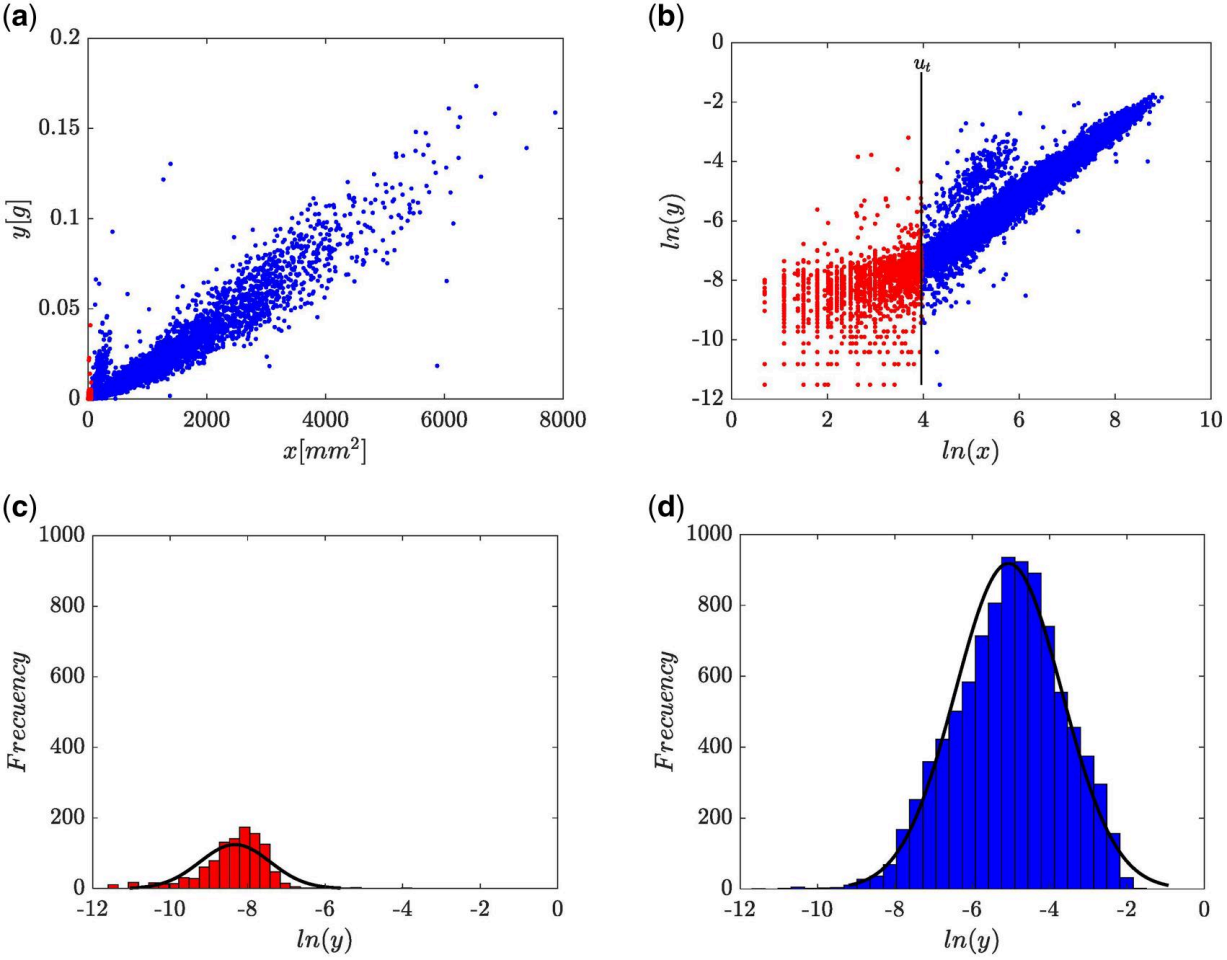
Exploratory graph assessment of variability of available eelgrass leaf biomass and related area measurements. Both (a) and (b) suggests differentiation in trends and in heteroscedasticity. (c) and (d) One to one, histograms of separate normal distributions fitted to red and blue data pools shown in (b).

For present data, Fig. 2a, readily hints at a possible size-related differentiation in the ensuing allometric linkage. Figure 2a also reveals a regular heteroscedastic pattern beyond the shown domed-like region around the smaller leaves. This spread suggests a leaf size differentiation in patterns of heteroscedasticity. Figure 2b, displays corresponding spread in log-scales $(\left(u,v\right)=\;\left(ln(x),ln(y\right)).$ It is worth pointing out that this transformation device is addressed only to gain clues on the complexity of the allometric protocol required to analyze present data, not for model identification aims. Indeed all model identification tasks were confined to the direct arithmetical scales of data and relied on maximum likelihood approaches. Looking at the plot in Fig. 2b, we can pick a threshold $u_{t}$ that places around u=4.0. Points that locate to the left of $u_{t}$ identify what we call the set of smaller leaves, while remaining ones pinpoint the set containing moderate to large leaves in data. This arrangement suggests that a mean response to be should accommodate to a biphasic allometric pattern. Furthermore, dispersion around Fig. 2b enhances a perception of discrepant variance patterns among red $\left(u\;\leq \;u_{t}\right)$ and blue-colored data points $\left(u\;\geq \;u_{t}\right).$ Besides, Fig. 2b hints on that for $u\;\geq \;u_{t}$ variance of u values is monotonically decreasing. In turn, Fig. 2c and d support a judgment that while a normal distribution can be consistently fitted around the subsample of red-colored points, a different normal one would do to the spread associated with blue ones. Altogether Fig. 2c and d hint at a weighted composite of two normal distributions as a candidate model for the distribution of leaf biomass as a random variable. In summary, spread plots in Fig. 2 suggest that while analyzing present data, a regression protocol involving a biphasic systematic part and bearing a multiplicative error composing a normal mixture distribution plus a size-related heteroscedasticity may render necessary.

### Fitting results

It is worth emphasizing that in the cases below in no way did we compromise the adequacy of fit to the use of logarithmic transformations. We reiterate that logarithmic transformations were only devices aimed at guidance. Supplementary Tables S4–S8 present fitting statistics of the protocols analyzed below. In all presented cases parameter estimation tasks depended on maximum likelihood procedures. Involved R codes are available from the corresponding author.

### Fitting results for the (*EMEM*, *w_H_*, 1, *N*) protocol

**Figure 3.**
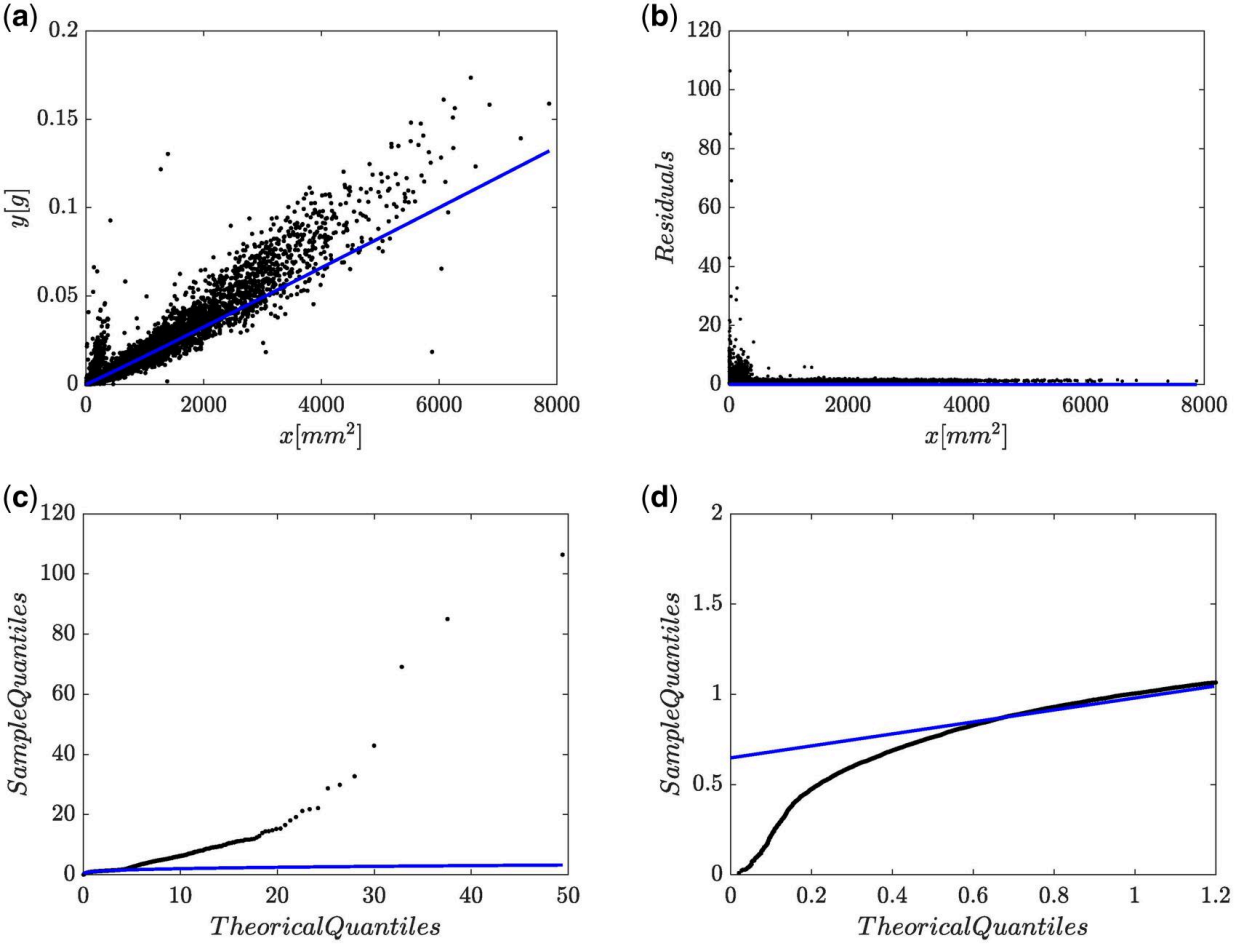
Spread plots of the fit of a ($EM,w_{H},1,N$) protocol. (a) Point spread around mean response function. (b) Residual plot. (c) Q–Q lognormal diagram. (d) Leftmost sector of Q–Q lognormal plot.

We first analyze data by relying on the ($\mathrm{EMEM},w_{H},1,N$) protocol, that is, the characterization of the EMEM arrangement coinciding with Equation (2). The associating fitting statistics are available from Supplementary Table S4. This fit reported an Akaike Information Criterion (AIC) value of AIC =-94745.76 [29]. Figure 3a shows a slightly downward bias of the mean response $\mu M\left[w_{H},1,N\right]\left(y|x\right),$ as given by Equation (7). Besides, Fig. 3b displays an uneven residual spread which suggests that $w_{H}(x,p)$ does not bear a suitable candidate for the systematic part. Figure 3c shows the corresponding Q–Q lognormal plot. Figure 3c and d reveals that the patterns in the extremes of the Q–Q lognormal plot depart from that expected; namely, we have heavy tails. These questions the ϵ∼N(0,σ) assumption concomitant to a MEM arrangement. Therefore, as suggested by the spread plots in Fig. 3 the fitting of Equation (2) to present data turns out to be inconsistent.

### Fitting results for the (*AEM* – *BP*, *w_H_N)* scheme

**Figure 4.**
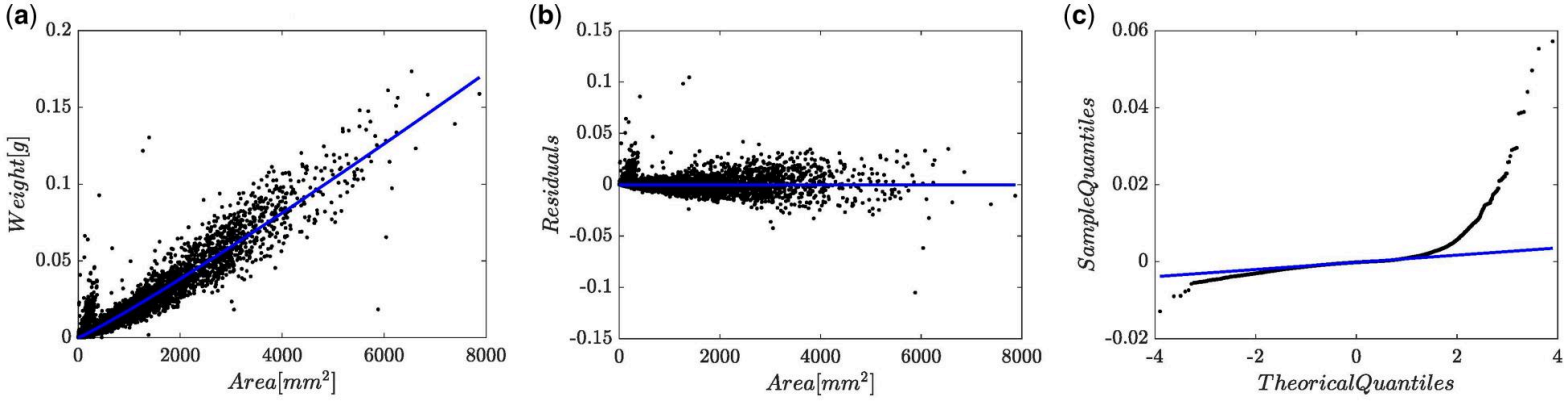
Spread graphs resulting from the fitting of the Breusch–Pagan modification of the additive error model of Equation (4) to available eelgrass leaf biomass and area data. In present framework ensuing fit identifies by the 4-tuple: $\left(\mathrm{AEM}-BP,\;w_{H},\;N\right)$ or by the DNLR-BP composite. Panel (a) spread about mean response function $\mu EA[w_{H},\;1,\;N]\left(y|x\right).$ Panel (b) residual plot. Panel (c) QQ-normal diagram. Heavy tails appear through a markedly asymmetrical pattern, which refers to an overall inconsistent $\left(\mathrm{AEM}-BP,\;w_{H},\;N\right)$ fit.

For comparison aims we also consider an AEM arrangement conforming to Equation (4), that is an allometric model composing a Huxley’s form systematic term and with additive errors being normally distributed. Recall that in the elaboration around Equation (4), we suggested using the DNLR-BP composite to account for heteroscedasticity. Considering the pattern learnt from Fig. 1a, we fitted the DNLR-BP scheme of Equation (4) with $\sigma (y|x)=\;\sigma \left(1\;+\;kx\right)$. Estimated parameters, and related statistics associating to a fit of the $\left(\mathrm{AEM}-BP,\;w_{H}\;N\right)\;$scheme on present data are available from Table S5 in the Supplementary Materials section. This $\left(\mathrm{AEM}-BP,\;w_{H},\;N\right)$ fit delivered an AIC value of AIC =-84528.9, which turns out to be greater than an AIC value of AIC =-94745.76 associating to the ($\mathrm{EMEM},w_{H},1,N$), (c.f. Table S4 in the Supplementary materials section). This result already favors the selection of the error structure hosting an ($\mathrm{EMEM},w_{H},1,N$) arrangement over the additive error one supporting the $\left(\mathrm{AEM}-BP,\;w_{H},\;N\right)$ triplet. Spread plots resulting from a $\left(\mathrm{AEM}-BP,\;w_{H},\;N\right)$ fit on present data appear in Fig. 4. Figure 4a presents the spread around the fitted mean response function $w_{H}(x,p)$. We can assess that even though a deviation from central tendency clearly displays for smaller leaves, dispersion about the mean response curve matches heteroscedasticity. Figure 4b portrays residual dispersion against projected response values showing a relative fairness about the zero lines. But still, Fig. 4c displays an asymmetrical heavy tails pattern for the error term. Portraying Q–Q Normal plot conceivably explains the fact, that even by appointing a Breusch–Pagan [28] variance function form, the ensuing protocol failed to deliver a good model for the real heteroscedastic spread conforming to present data. We can also be aware that the central portion of the spread fits properly to a normal distribution, but extremes have a decidedly different spread than expected for that distribution; that is, we can be ascertained of a heavy tails pattern. We can corroborate the existence of such an outline by assessing the value of the kurtosis coefficient. We obtained kurtosis =14.176, which happens to be much greater than the value corresponding to a normal distribution (kurtosis=3.0). Therefore, agreeing to Wheeler [30], a heavy tails pattern validates. In other words, heavy tails appear through a markedly asymmetrical pattern, which refers to an overall inconsistent $\left(\mathrm{AEM}-BP,\;w_{H},\;N\right)$ fit.

### Fitting results for the (*EMEM, w_BH_*, 1, *N*) protocol

**Figure 5.**
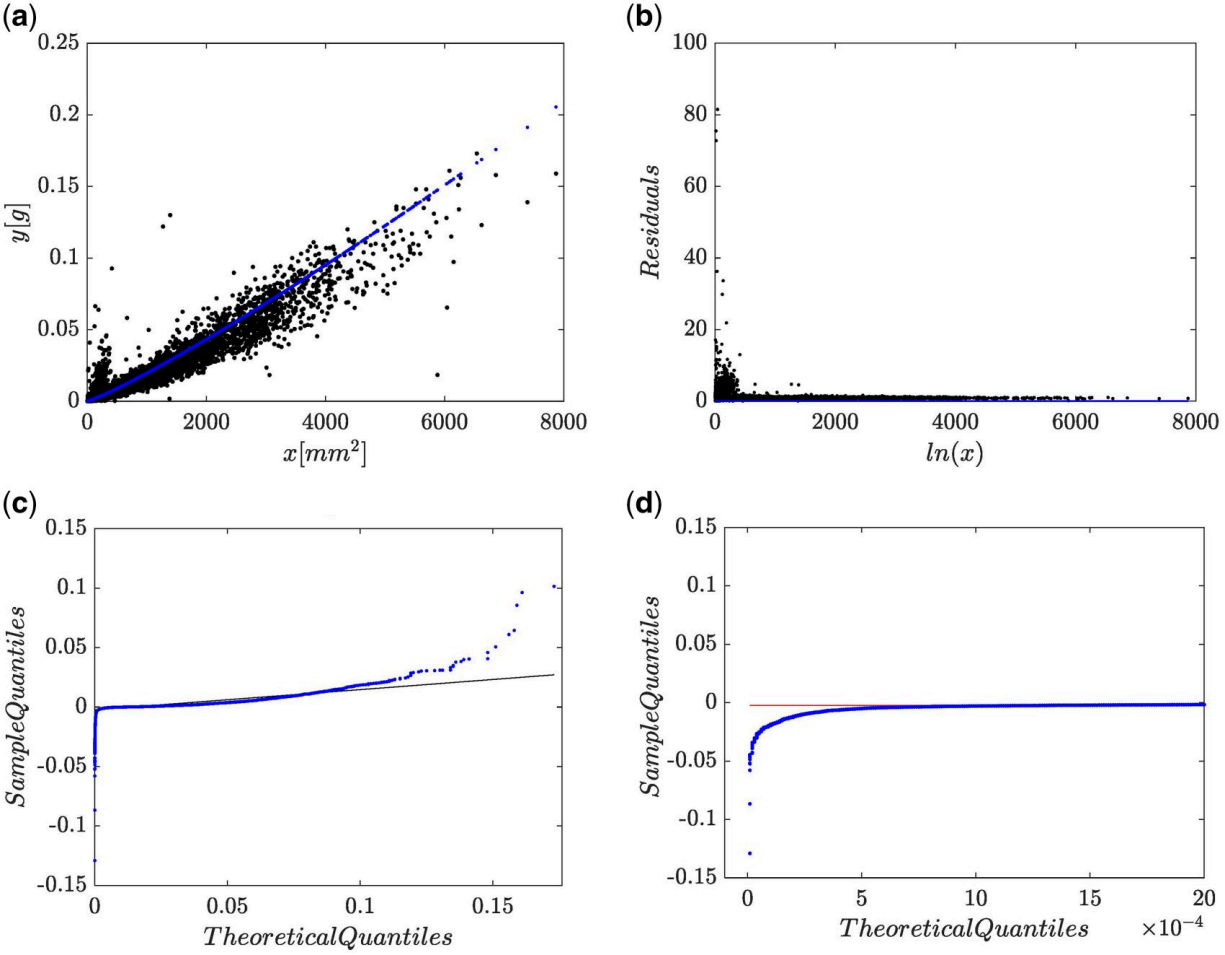
Spread plots associating to a $(\mathrm{EMEM},\;w_{BH},\;1,\;N)\;$arrangement. (a) Spread about the mean response function. (b) Residual plot. (c) Q–Q lognormal diagram with its leftmost sector showing in (d).

We now go over the fit of the $(\mathrm{EMEM},\;w_{BH},\;1,\;N)\;$arrangement, entailing the EMEM of Equation (5) composing $w_{BH}(x,p)$ as given by Equation (8) but maintaining $\epsilon ∼N(0,\sigma^{2}$). Related fitting statistics are included in Supplementary Table S6. This fit resulted on an AIC value of AIC=-96385.61. Fitted value of the breakpoint resulted on $x_{b}=3.082518e\;+\;01$. Figure 5 displays corresponding spread plots. Compared to Figure 2a, spread about $w_{BH}(x,p)$ improved (Fig. 5a). Nevertheless, concerning residual distribution an uneven spread pattern maintains (Fig. 5b). Figure 5c displays the Q–Q lognormal diagram. Fig 5d shows the leftmost Q–Q lognormal plot sector. Fig. 5c along Fig. 5d, uphold an asymmetric heavy tails pattern. Therefore, in the settings of the EMEM of Equation (5) and present data, enhancing to $w_{BH}\left(x,p\right)$, while holding to $\epsilon ∼N(0,\sigma^{2}$) does not grant a consistent fit.

### Fitting results for the (*EMEM, w_BH_*, 1, *NM*_2_) composite

**Figure 6.**
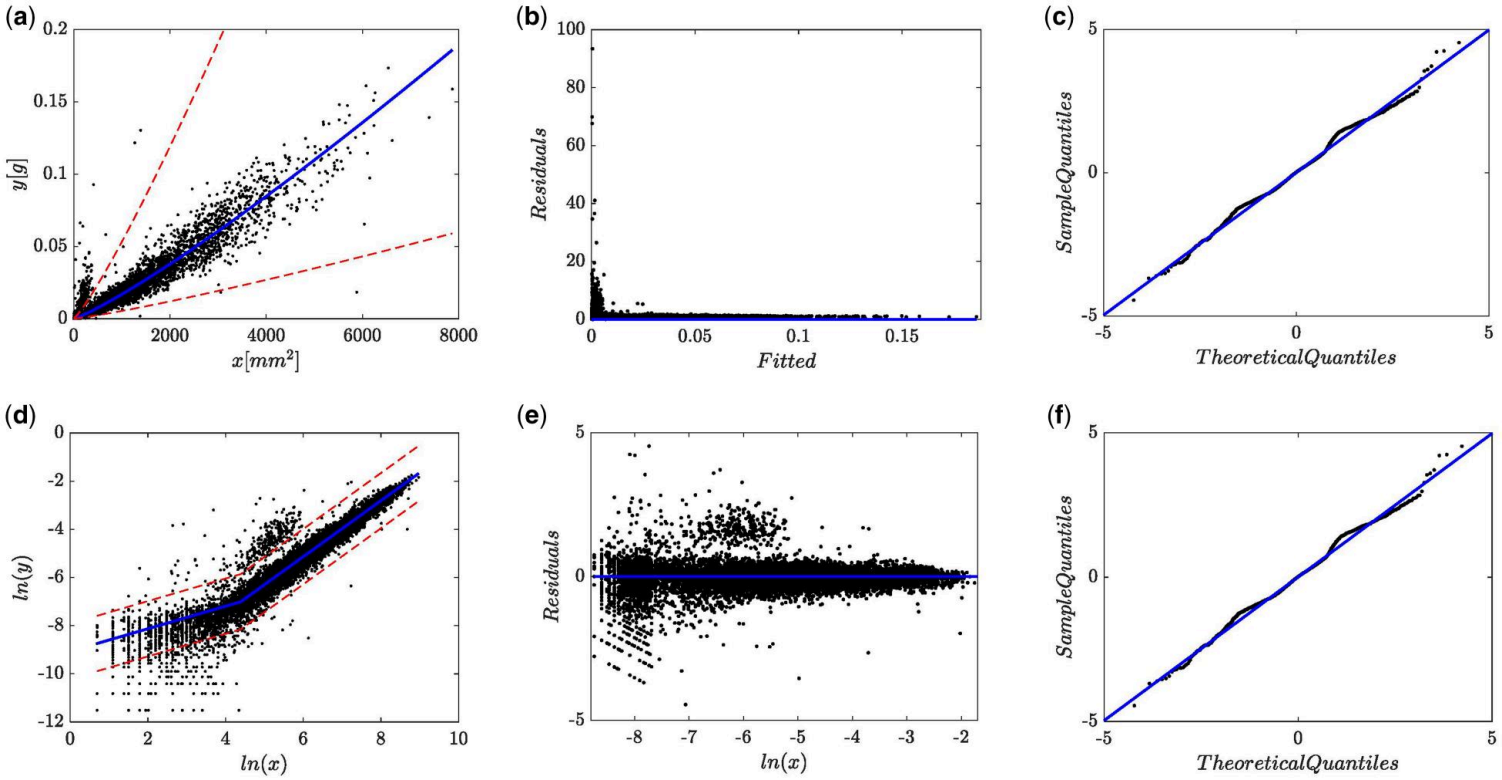
Spread plots associating to the $(\mathrm{EMEM},\;w_{BH},1,{NM}_{2})\;$scheme. (a) Spread about the mean response function. (b) Residual dispersion. (c) Q–Q LogN2M plot. (d) Spread about the ELM produced mean response. (e) Residual dispersion. (f) Q–Q N2M plot.

The insight provided by plots in Figure 2 plus the inconsistencies of fits performed so far paved the way to consideration of a further extension of the complexity in the EMEM of Equation (5). The fit of a $\left(\mathrm{EMEM},\;w_{BH},1,{NM}_{2}\right)$ arrangement produced the fitting statistics included in Supplementary Table S7. Identified break point was $x_{b}=4.474265e\;+\;01$. The AIC value resulted in AIC=-101783.8. This AIC value turns to be smaller compared to previously fitted protocols (Supplementary Table S4 through Table S6). Figure 6a presents the spread about the mean response function $w_{BH}(x,p)$. The accompanying 95% confidence strip portraits anomaly wider. Figure 6b shows residual dispersion and Fig. 6c displays joining Q–Q LogN2M plot. Opposing to Figure 3c,d, 4c, and Figure 5c,d, we do not have heavy tails. For comparison aims Figure 6d offers the spread about the mean response function $ln{(w}_{BH}(x,p$)), associating to the accompanying $\left(\mathrm{ELM},\;w_{BH},1,{NM}_{2}\right)$ arrangement. This fit identified a value $u_{b}=3.98$ in geometrical space, that corresponds to the break point $x_{b}$ in direct scales. This plot reveals than on added complexity corroborates it reasonable holding to the observation from Figure 2b, that for $u\;\geq \;u_{t}$ variance of u values is monotonically decreasing. Figure 6e shows residual dispersion in geometrical scales and Fig. 6f displays joining Q–Q N2M plot associating to the $\left(\mathrm{ELM},\;w_{BH},1,{NM}_{2}\right)$ fit.

### Fitting results for the (*EMEM w_BH_, h_p_* (*x,c*), *NM*_2_) compound

Plot analysis so far points that while envisaging the sought enhancement, both the statement ${w(x,y)=w}_{BH}(x,y)$ and the randomness assumption $\epsilon ∼{NM}_{2}\left(0,s\right)$ should maintain. But we still need to revise the inconsistent wideness of the 95% confidence strip shown in Figure 6a. Recalling Figure 2b, we can learn that the variance of response replicates at a covariance level switches patterns about the breakpoint${\;u}_{t}$. It then turns intuitively reasonable that adding complexity as required mainly circumscribes to adapting the form of the variance scaling function $h\left(x,c\right)$. Thus, we propose $h\left(x,c\right)$ being expressed in a piecewise form $h_{p}\left(x,c\right),\;$namely,

(9) $$h_{p}\left(x,c\right)=\left\{\begin{array}{c} 1,\;if,\;0\;<\;x\;\leq \;x_{b}\;\\ {\left(\frac{x_{b}}{x}\right)}^{c},\;if,\;x_{b}\;<\;x \end{array}\right.,$$

where $x_{b}$ is the breakpoint associating to $w_{BH}\left(x,y\right)$ (c.f. Equation (8)). Concomitantly the composing error function $\psi \left(x,\epsilon \right)$ (c.f. Equation (6)) defines by $\psi \left(x,\epsilon \right)=\exp\left(h_{p}\left(x,c\right)\;\epsilon \right).$

**Figure 7.**
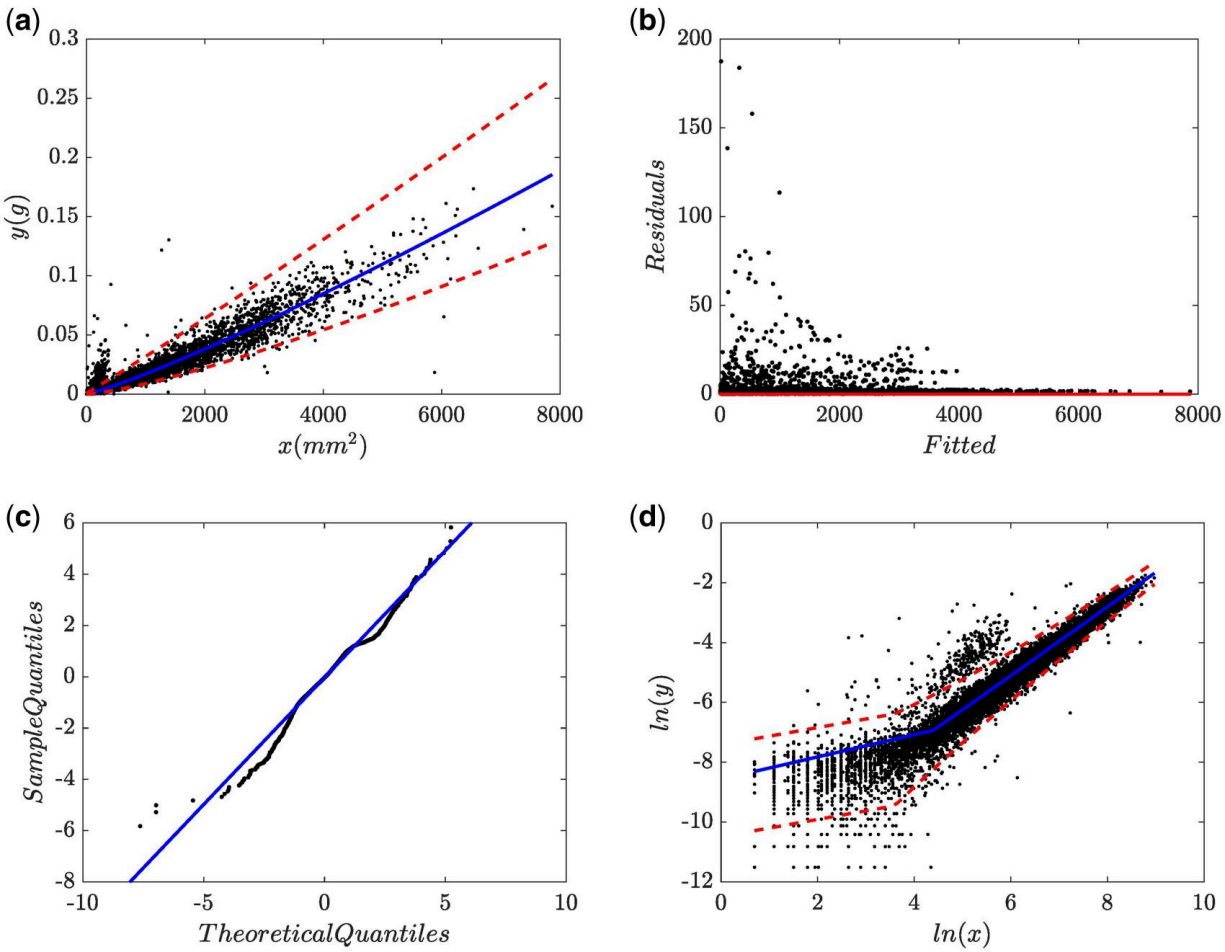
Spread plots supplementary of a$\;(\mathrm{EMEM},\;w_{BH},h_{p},{NM}_{2})$ fit. (a) Spread about fitted mean response. Dashed lines bound a 95% confidence strip. (b) Residual plot. (c) Q–Q normal mixture plot. (d) Dispersion about the piecewise linear mean response function in geometrical space, along with corresponding 95% confidence strip.

Fitting statistics of the resulting $\left(\mathrm{EMEM},\;w_{BH},h_{p}(x,c),{NM}_{2}\right)$ arrangement summarize in Supplementary Table S8. Figure 7 pictures relating spreads. Present scheme produced AIC=−103205.6 leading to a difference ΔAIC=1421.8, when compared to the $(\mathrm{EMEM},\;w_{BH},1,{NM}_{2})\;$fit (Supplementary Table S7.). Distribution of observed values around fitted mean response function shown in Fig. 7a portraits unbiased and fairer in comparison to the $\left(\mathrm{EMEM},\;w_{BH},1,{NM}_{2}\right)$ set up (Figure 6). Figure 7b shows residual spread. Figure 7c shows relating Q–Q lognormal mixture plot. In turn, the 95% confidence region around the biphasic mean response displaying in Fig. 7a improved compared to the exaggeratedly wide-open arrangement shown in Fig. 6a. Besides, the 95% confidence strip shown in Fig. 7d consistently agrees to the piecewise-like heteroscedastic spread conjectured from Fig. 2b.

### Comparison of the EMEM to existing models

Table 1 below allows an AIC and concordance correlation coefficient (CCC) [31] based model performance comparison of (i): the new full complexity scheme $\left(\mathrm{EMEM},\;w_{BH},\;h_{p}\left(x,c\right),{NM}_{2}\right)$, that is, the extension of the ordinary Huxley’s based multiplicative allometric protocol to allow a biphasic mean response, log-mixture of normal distribution error and a piecewise-like heteroscedasticity, (ii): $\left(\mathrm{EMEM},\;w_{BH},\;1,\;N\right)$, the conventional multiplicative allometric scheme but extended in order to consider a biphasic systematic part but keeping lognormal errors, and (iii): $\left(\mathrm{EMEM},\;w_{BH},1,{NM}_{2}\right),$ the customary multiplicative error model extended to a biphasic systematic term and a log-mixture of normal distributions error, and the existing models (iv): ($\mathrm{EMEM},w_{H},1,N$), the regular Huxley power function multiplicative error model which assumes lognormal errors, (v): $\left(\mathrm{AEM}-BP,\;w_{H},\;N\right),$ the conventional additive error model with a systematic term expressed in a Huxley’s power function form and Breusch–Pagan adapted heteroscedastic errors, (vi): $\left(\mathrm{AEM},\;w_{H}\;+\;c,\;N\right)$, standing for the three parameter power function with normally distributed. It is worth stating that opposite to the $\left(\mathrm{AEM},\;w_{H}\;+\;c,\;N\right)$ scheme the $(\mathrm{AEM}-BP,\;w_{H},\;N$) is a suitable candidate to analyze allometric settings that pass through the origin, which concerns to present data. In any event, we include the $\left(\mathrm{AEM},\;w_{H}\;+\;c,\;N\right)$ only for comparison of present model to existing schemes, (vii): $\left(\mathrm{EMEM},\;w_{H},\;1,\;{NM}_{2}\right)$ the multiplicative allometric scheme based on Huxley’s power function but extended in order to consider a log mixture of normal distributions error, fitted by Villa-Diharce et al. [29].

**Table 1.** AIC and CCC-based comparison of the EMEM to existing protocols.

Model	AIC	ΔAIC	CCC
$\left(\mathrm{AEM},\;w_{H}+c,\;N\right)$	−41985.6	61 220	0.9581
$\left(\mathrm{AEM},\;w_{H},\;N\right)$	−84528.9	18 676.7	0.9575
($\mathrm{EMEM},w_{H},1,N$)	−94745.76	8423.84	0.9232
$(\mathrm{EMEM},\;w_{BH},1,\;N)\;$	−96385.61	6833.99	0.9520
$(\mathrm{EMEM},\;w_{H},1,\;{NM}_{2})\;$	−99520.85	3635.75	0.9550
$\left(\mathrm{EMEM},\;w_{BH},1,{NM}_{2}\right)$	−101783.8	1421.8	0.9581
$\left(\mathrm{EMEM},\;w_{BH},\;h_{p},{NM}_{2}\right)$	−103205.6	0	0.9581

AIC and CCC values in Table 1 support selecting the new model $\left(\mathrm{EMEM},\;w_{BH},\;h_{p},{NM}_{2}\right)$ over the remaining ones. Particularly the three-parameter power function $\left(\mathrm{AEM},\;w_{H}\;+\;c,\;N\right)$, displayed the weakest fitting capabilities. Moreover, the value c=0.000293 fitted by $\left(\mathrm{AEM},\;w_{H}\;+\;c,\;N\right)$ on raw data implies the three-parameter power function assigning positive biomass to a leaf of a zero area. Moreover, $\left(\mathrm{AEM},\;w_{H}\;+\;c,\;N\right)$ fitted c=-0.000250 for the processed data set, which projects negative biomasses for suitably small leaf areas. This is aberrant since data cleaning reduced variability in raw data, so a more consistent fit could be expected. Detected inconsistencies are explained by the fact that the spread in Fig. 2a already suggests that a protocol bearing allometry through the origin should be used when attempting to model present data.

### Comparison of the reproducibility strength of EMEM-derived allometric proxies for eelgrass leaf biomass

For comparison aims, in Supplementary Table S9 we provide statistics for the valuation of the reproducibility power of EMEM-derived allometric proxies for observed monthly average leaf biomass in shots reported in the present data. In Supplementary Table S9, we make available AIC, Standard Error of Estimate, and Mean Prediction Error [32–35]. Similarly, we bring in Lin’s CCC, denoted here through the CCC symbol [31, 36]. We also propose a Relative Absolute Deviation (RAD) index value [29]. To calculate these statistics, we first obtain the absolute deviation between the overall mean of monthly averages of observed leaf biomass values and the corresponding one derived allometrically, then we divide by the overall mean of monthly averages of observed individual leaf biomass values. Statistics included in Supplementary Table S9 corroborate the outmost reproducibility strength offered by the $(\mathrm{EMEM},\;w_{BH},h_{p}\left(x,c\right),{NM}_{2})\;$scheme.

## Summary

Here, we analyzed eelgrass leaf biomass and area data exhibiting a marked size-related heterogeneity. This is patent by looking at the small cloud of blue points separated from the larger group (Fig. 2b). The presence of these replicates perhaps explains a lack of systematization at data gathering. Over-dispersion precluded adequacy of a MEM approach, suggesting processing data aimed to remove unduly replicates. Nevertheless, as we previously reported, achieving regularity to Huxley's power function-like trend required the removal of many replicates, thereby questioning the integrity of a data-cleaning approach [29]. Then, here, we adapted the complexity of the MEM setup’s error term, resulting in the EMEM alternate. In contrast to the conventional MEM, the complex allometry constructs backing the EMEM adaptation could reliably identify a biphasic model-like systematic part masked by variability in data. Moreover, the detected break point in Zostera marina could be interpreted as a threshold beyond which plants assign to leaves a relatively more significant amount of tissue to resist drag force effects that induce damage and separation from shoots. Achieving the EMEM suitability also relied on an error term conforming to a weighted Normal Mixture distribution. According to plots in Figure 7, it delivered a coherent Q–Q lognormal mixture spread and a remarkable reproducibility strength of derived proxies (Supplementary Table S9). By offering an adaptation of Huxley’s original theory, the present approach enables substantiating non-destructive allometric proxies aimed at eelgrass conservation. Furthermore, the viewpoint endorsed here could also make data cleaning unnecessary. We consider that presently offered EMEM sustains a suitable model for other instances of allometric examination where randomness nurturing the error term falls outside normality [29, 37], and the pattern of heteroscedasticity accommodates into a piecewise-like form.

## Conclusion

Present results demonstrate that enhancing the complexity of the systematic relationship while keeping normality of the random variable ϵ in the model of Equation (2), does not necessarily grant a suitable fit. We previously explained the advantages of considering distributions other than normal for the random variable ϵ [29, 37]. But present results show that extending the complexity of both the systematic part and error term linking Equation (2) as to allow non-normality of the random variable ϵ, plus consideration of a mixed form of the heteroscedastic design could credit for overall fit improvement. It becomes pertinent to stress that projections of the allometric response are highly sensitive to error propagation of parameter estimates [38]. Therefore, as we have demonstrated here, in the allometric examination, it would be pertinent to recall the advantages of the presently offered EMEM arrangement to achieve overall model suitability, thereby granting accuracy in deriving leaf biomass projections.

From a qualitative perspective, some last remarks remain. Indeed, in examining bivariate allometric data, we can find a wide variety of dispersion patterns, which explains the adoption of models, including complex shapes for the systematic part and non-normal distributions generating the error term [13, 29, 39–42]. However, it should be noted that the pattern of variation in the data examined here presents analytical subtleties that demand addressing schemes beyond the traditional complex allometry ones. Indeed, the dispersion cloud observed at the beginning of Fig. 2b explains the modeling challenge we refer to. Definitely, for leaf dry weight and the corresponding area in Zostera marina, the pattern observed in Fig. 2b, could not be modeled, for instance, by relying upon an allometric scheme that does not pass through the origin claimed by Sartori and Ball [16], this because to a leaf of zero area It there must correspond a vanishing dry weight. Upon this, given the problem posed by the anomalous dispersion around Fig. 2b, conventional modeling approaches might suggest resorting to data cleaning. However, as we have explained [29], achieving the consistency of the traditional Huxley multiplicative error scheme would require eliminating many replicates, making such a try a questionable procedure. Given this circumstance, the usefulness of the EMEM that we propose here stands out. Indeed, the inclusion of different patterns of heteroskedasticity made it possible to consistently identify the through-the-origin allometric pattern that is expected for the data analyzed without depending on data cleaning techniques. Overdispersion at the pool of small leaves could be explained by a lack of standardization at data gathering since reduced leaf sizes could prompt measurement errors tied to the weight-scale device. However, despite noise-inducing effects, the complexity added by the presently offered EMEM was advantageous over conventional schemes that failed to provide consistent fits given the present data.

## Supplementary Material

bpae024_Supplementary_Data

## Data Availability

No new data were generated or analyzed in support of this research. Data will be available from the corresponding author provided a faire usage agreement. Data will be available from the corresponding author provided a faire usage agreement.

## Supplementary data

Supplementary files available at *Biology Methods and Protocols* online.
