# Supplementary material for: An extended multiplicative error model of allometry: Incorporating systematic components, non-normal distributions, and piecewise heteroscedasticity
Source: Biol Methods Protoc. 2024 Apr 18;9(1):bpae024. doi: 10.1093/biomethods/bpae024 (PMC11099667; doi:10.1093/biomethods/bpae024)
Supplement: bpae024_Supplementary_Data [file bpae024_supplementary_data.docx]

## Supplementary materials

## Exploratory analysis of raw and processed data sets

Table 1 describes the distribution pattern, in terms of quantiles, for a sample of 10412 measurements of eelgrass leaf weights and related areas taken over 13 months conforming to the present raw data. The first four columns in the uppermost row label the minimum followed by quantiles of probability 0.1, 0.25, 0.50. Correspondingly, the fifth column in the first row presents the sample mean followed by quantiles of probability 0.75 and 0.90 before the maximum. The second row presents leaf dry weight values ($y)$ third row associates to corresponding areas$x$. The third and fourth rows present transformed $ln(y)$ and $ln(x)$ values one-to-one. Similarly, Table 2 shows the variation pattern of the 7840 observations resulting after applying to raw data the quality control procedure described in [29, 38] designed to remove replicates considered significantly discrepant from the mean response function of a simple allometric model for a leaf dry weight response in terms of a leaf area covariate.

**Table 1**. **Data Distribution Pattern, Explained in terms of Quantiles**. Values of minimum, maximum, sample mean, and quantiles for measurements of eelgrass dry weight$(y\left[ g \right])$ and area $(x\left[ \mathrm{mm}^{2} \right])$ (and their logarithms) of a sample of 10412 leaves before applying a data cleaning procedure aimed to eliminate outliers.

| **Variable** | **Min** | **0.10** | **0.25** | **0.50** | **Mean** | **0.75** | **0.90** | **Max** |
| --- | --- | --- | --- | --- | --- | --- | --- | --- |
| $\boldsymbol{y}\left[ \mathbf{g} \right]$ | 0.00001 | 0.00042 | 0.00154 | 0.00564 | 0.01293 | 0.01477 | 0.035443 | 0.38058 |
| $\boldsymbol{x}\left[ \mathbf{mm}^{\mathbf{2}} \right]$ | 2.00 | 32.50 | 127.5 | 355.2 | 690.5 | 836.0 | 1859.00 | 7868.0 |
| $\boldsymbol{ln(y)}$ | -11.513 | -7.7753 | -6.4760 | -5.1779 | -5.4001 | -4.2152 | -3.33983 | -0.9661 |
| $\boldsymbol{ln(x)}$ | 0.6931 | 3.48124 | 4.8481 | 5.8728 | 5.6729 | 6.7286 | 7.527794 | 8.9706 |

**Table 2. Variation Pattern of Data After Applying Quality Control Procedures.** Values of minimum, maximum, sample mean, and quantiles for measurements of eelgrass dry weight$(y\left[ g \right])$ and area ($x[\mathrm{mm}^{2}])$ (and their logarithms) of a sample of 7840 leaves remaining after applying a data cleaning procedure aimed to eliminate outliers.

| **Variable** | **Min** | **0.10** | **0.25** | **0.50** | **Mean** | **0.75** | **0.90** | **Max** |
| --- | --- | --- | --- | --- | --- | --- | --- | --- |
| $\boldsymbol{y}\left[ \mathbf{g} \right]$ | 0.00001 | 0.00042 | 0.00155 | 0.00581 | 0.01435 | 0.01668 | 0.04157 | 0.38058 |
| $\boldsymbol{x}\left[ \mathbf{mm}^{\mathbf{2}} \right]$ | 2.0 | 35.90 | 140.0 | 395.8 | 781.2 | 990.0 | 2171.00 | 7868.0 |
| $\boldsymbol{ln(y)}$ | -11.513 | -7.7753 | -6.4695 | -5.1482 | -5.3214 | -4.0935 | -3.1803 | -0.9661 |
| $\boldsymbol{ln(x)}$ | 0.6931 | 3.58070 | 4.9416 | 5.9808 | 5.7837 | 6.8977 | 7.68294 | 8.9706 |

Comparing quantile values for the leaf dry weight ($y$), and corresponding area ($x$) variables reported in Tables 1 and 2 for crude and processed data respectively, we can ascertain that despite removing many discrepant observations by data cleaning, both original and remnant sets show an equivalent distribution pattern. This similarity in the distribution before and after data processing can be better perceived in the Q-Q (quantile-quantile) graphs observed in Fig. 1, for raw (Fig. 1A) and processed data (Fig. 1B) respectively.

**
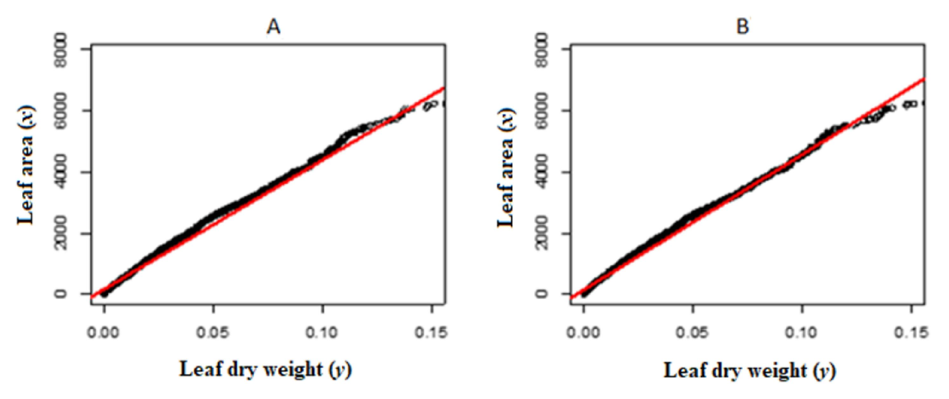
**

**Figure 1**. **Q-Q (Quantile-Quantile) Graphs for Raw and Processed Data.** Q-Q plots (quantile-quantile) comparing distribution patterns, of observations of eelgrass leaf area$x$ and associated dry weight $y$, for raw in (A) and the processed data in (B).

Q-Q plots in Fig. 1 compare the distributions (regardless of what they are) of the leaf area$x$ and associated dry weight $y$ variables. The linear relationship observed between the quantiles of both variables underline that both variables have similar distributions, although with different parameters. This similarity between distributions of both variables is observed for both sets of observations. The great similarity of the fitted straight lines for both sets of observations in Table 3 confirms that the referred distribution pattern does not change after data processing.

**Table 3. Statistics of Straight Lines Fitted to Raw and Processed Data.** Parameter estimates of straight lines fitted on raw and processed data and shown in the Q-Q plots of Fig. 1.

| **Data set** | **Estimate** | **Standard Error** |
| --- | --- | --- |
| **Raw** |  |  |
| Intercept | $-1.16\times{10}^{-3}$ | $8.95\times{10}^{-5}$ |
| slope | $2.04\times{10}^{-5}$ | $7.84\times{10}^{-8}$ |
| **Processed** |  |  |
| Intercept | $-1.82\times{10}^{-3}$ | $9.03\times{10}^{-5}$ |
| Slope | $2.07\times{10}^{-5}$ | $7.13\times{10}^{-8}$ |

**
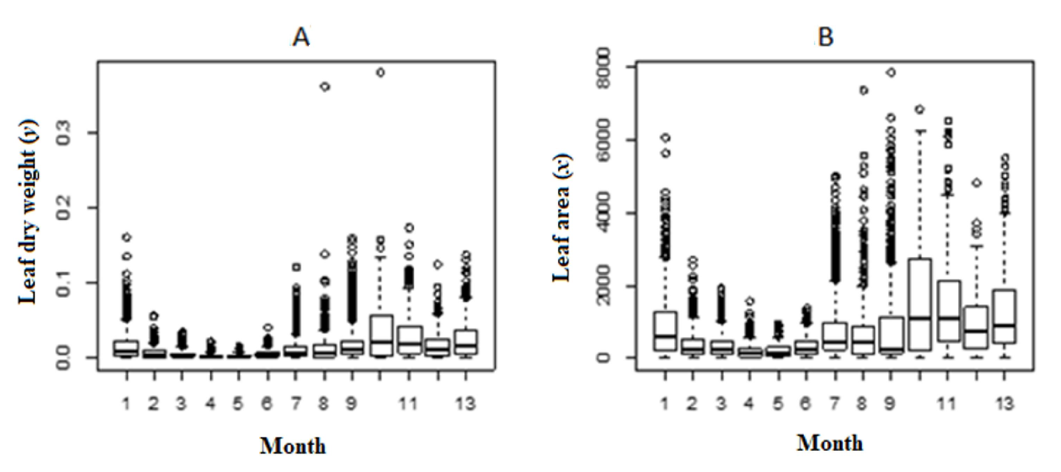
**

**Figure 2**. **Boxplots for raw leaf dry weight and area values.** Boxplots for values of eelgrass leaf dry weight $y$ in (A) and linked area$x$ in (B) classified by month, as indicated in the horizontal axis. The data is from a sample of 10412 observations, before applying data processing.

**
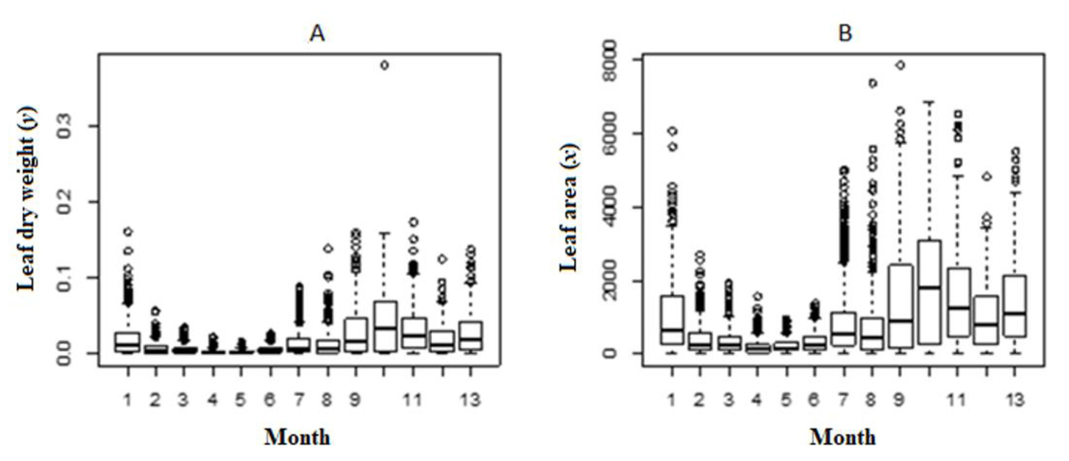
**

**Figure 3.** **Boxplots for processed leaf dry weight and area values**. Boxplots for values of eelgrass leaf dry weight $y$ in (A) and linked area$x$ in (B) classified by month, as indicated in the horizontal axis. The data is from a sample of 7840 observations, remaining after applying an outlier removal procedure.

Figure 2 and Figure 3 display boxplots for the 13 months long sampling scheme for raw and processed data one to one. We can learn that for raw data, from month 2 to month 6, a reduction in the values of both leaf dry weight and linked area occurred. This is perhaps due to an increase in temperature during those months. Processed data exhibits similar dynamics through time for these variables. Moreover, the overall variation patterns of raw and processed data, throughout the 13 months of sampling, are similar. This even though data processing eliminated a significant number of discrepant observations (23%). This perhaps could be explained by a lack of standardization of data gathering routines.

## Fitting statistics associated with the regression protocols included in the results section

### Fitting statistics for the ($\boldsymbol{EMEM,}\boldsymbol{w}_{\boldsymbol{H}}\boldsymbol{,1,N}$) scheme

The regression scheme of Equation (2) can be put up as a particular case of the presently offered EMEM, typified through Equation (4), by setting a Huxley’s power function driven systematic term, that is, $w\left( x,p \right)=w_{H}(x,p)$, a variance scaling function $h\left( x,c \right)=1,$ and a basic error getting random variable $\epsilon\sim N(0,\sigma^{2}$). Therefore, the model of Equation (2) given by the 4-tuple: ($EM,w_{H},1,N$), which bears a Huxley’s power function borne allometric model with multiplicative errors being lognormally distributed. Fitting statistics are presented in Table 4 bellow.

**Table 4. Estimated parameters, and related statistics associating to a (**$\boldsymbol{EMEM,}\boldsymbol{w}_{\boldsymbol{H}}\boldsymbol{,1,N}$**) scheme.** We include parameter estimates with their projected uncertainties, t-value, p-value, LogLikMx and AIC stand for Maximum Loglikelihood and Akaike information index values one to one.

| **Parameter** | **Estimate** | **Std. Err.** | **Confidence Interval (95%)** | ***t*-value** | ***p*-value** |
| --- | --- | --- | --- | --- | --- |
| $\beta$ | 1.3543e-05 | 2.8836e-07 | (1.2978e-05, 1.4108e-05) | 47.0 | $<1.0\times{10}^{-30}$ |
| $\alpha$ | 1.0239e+00 | 3.6235e-03 | (1.0168e+00, 1.0310e+00) | 282.5 | $<1.0\times{10}^{-30}$ |
| $\sigma$ | 5.6609e-01 | 3.9231e-03 | 5.5840e-01, 5.7378e-01) | 144.3 | $<1.0\times{10}^{-30}$ |
| logLikMx | 47375.9 |  |  |  |  |
| AIC | -94745.76 |  |  |  |  |

### Fitting statistics for the ($\boldsymbol{AEM,}\boldsymbol{w}_{\boldsymbol{H}}\boldsymbol{,N}$) scheme

For comparison aims we consider an AEM as entailed by Equation (4), that is an allometric model composing Huxley’s form systematic term and with additive errors being normally distributed We refer to this protocol using the symbol **(**AEM, $w_{H},$ $N\boldsymbol{)}$. Recall that in the elaboration around Equation (4) we suggested using the DNLR-BP composite to account for heteroscedasticity. Considering the pattern learnt from Figure 1A, we fitted the DNLR-BP scheme of Equation (4) with $\sigma(y|x)=$ $\sigma\left( 1+kx \right)$. Estimated parameters, related statistics associating to a fit of the scheme on present data are available from Table 5 bellow.

**Table 5: Estimated parameters, related statistics associating to a (*AEM*,** $\boldsymbol{w}_{\boldsymbol{H}}$**,** $\boldsymbol{N}\mathbf{)}$ **scheme.** We include parameter estimates with their estimated uncertainties, t-value, p-value, LogLikMx and AIC stand for Maximum Loglikelihood and Akaike information index values one to one.

| **Parameter** | **Estimate** | **Std. Err.** | **Confidence Interval (95%)** | ***t*-value** | ***p*-value** |
| --- | --- | --- | --- | --- | --- |
| $\beta$ | 9.76e-06 | 3.76e-07 | (9.048e-06, 1.052e-05) | 26.0 | $<2\times10^{-16}$ |
| $\alpha$ | 1.0883 | 0.0053 | (1.078, 1.0987) | 205.73 | $<2\times10^{-16}$ |
| $\sigma$ | 0.002557 | 3.4042e-05 | (0.00249, 0.00262) | 75.2 | $<2\times10^{-16}$ |
| $k$ | 0.001215 | 4.1431e-05 | (0.001134, 0.001296) | 29.4 | $<2\times10^{-16}$ |
| logLikMx | 42268.43 |  |  |  |  |
| AIC | -84528.9 |  |  |  |  |

### Fitting statistics for the ($\boldsymbol{EMEM,}\boldsymbol{w}_{\boldsymbol{BH}}\boldsymbol{,1,N}$) composite

In order to contemplate an (EMEM, $w_{BH},1, N)$ arrangement in the settings of the EMEM of Equation (4) we let $w(x,p)$ acquiring a biphasic form, that is, $w\left( x,p \right)=w_{BH}(x,p)$ (c.f. Equation (6)), also, a variance scaling function $h(x,c)=1$ and keeping a normality assumption for the error driving random variable $\epsilon$, that is, $\epsilon\sim N\left( 0,\sigma\right)$. In summary. For $w_{BH}(x,p)$ as given by Equation (6) parameter set becomes $p=(\beta, \alpha$,$\lambda$, $x_{b})$ with $x_{b}$ identified as a breakpoint for transition from a first allometric phase $y=\beta{x^{\alpha}}$ holding on $x\leq x_{b}$ to a second one $y=\beta{x_{b}^{\alpha-\lambda}x^{\lambda}}$ working on $x>x_{b}$. Table 6 presents relating fitting statistics.

**Table 6. Fitting statistics associating to a (EMEM,** $\boldsymbol{w}_{\boldsymbol{BH}}\boldsymbol{,1, N}$**) scheme.** We include parameter estimates with their projected uncertainties, t-value, p-value, LogLikMx and AIC stand for Maximum Loglikelihood and Akaike information index values one to one.

| **Parameter** | **Estimate** | **Std. Err.** | **Confidence Interval (95%)** | ***t*-value** | ***p*-value** |
| --- | --- | --- | --- | --- | --- |
| $\beta$ | 1.008219e-04 | 6.363697e-06 | (8.835e-05,1.133e-04) | 15.84 | $<1.0\times{10}^{-30}$ |
| $\alpha$ | 3.416174e-01 | 2.454368e-02 | (2.935e-01, 3.897e-01) | 13.92 | $<1.0\times{10}^{-30}$ |
| $\lambda$ | 1.138851e+00 | 4.680635e-03 | (1.130e+00,1.148e+00) | 243.32 | $<1.0\times{10}^{-30}$ |
| $x_{b}$ | 3.082518e+01 | 1.212293e+00 | (2.845e+01,3.320e+01) | 25.43 | $<1.0\times{10}^{-30}$ |
| $\sigma$ | 5.231219e-01 | 3.625390e-03 | (5.160e-01, 5.302e-01) | 144.29 | $<1.0\times{10}^{-30}$ |
| logLikMx | 48197.81 |  |  |  |  |
| AIC | -96385.61 |  |  |  |  |

### Fitting results for the ($\boldsymbol{EMEM,}\boldsymbol{w}_{\boldsymbol{BH}}\boldsymbol{,1,}\boldsymbol{NM}_{\boldsymbol{2}}$) arrangement

In order to contemplate an (EMEM, $w_{BH},1, N)$ arrangement in the settings of the EMEM of Equation (4) we let $w(x,p)$ acquiring a biphasic form, that is, $w\left( x,p \right)=w_{BH}(x,p)$ (c.f. Equation (6)), also, a variance scaling function $h(x,c)=1$, but switching from a $\epsilon\sim N\left( 0,s \right)$ to a $\epsilon\sim{NM}_{2}\left( 0,s \right)$ assumptions. Table 7 gives the associating fitting statistics.

**Table 7.** **Fitting statistics associating to a (EMEM,** $\boldsymbol{w}_{\boldsymbol{BH}}\boldsymbol{,1,}\boldsymbol{NM}_{\boldsymbol{2}}$**) scheme**. We include parameter estimates with their projected uncertainties, t-value, p-value, LogLikMx and AIC stand for Maximum Loglikelihood and Akaike information index values one to one.

| **Param.** | **Estimate** | **Std. Err.** | **Conf.Int (95%)** | **t-value** | **p-value** |
| --- | --- | --- | --- | --- | --- |
| $\beta$ | 1.265613e-04 | 6.486187e-06 | (1.139e-04, 1.393e-04) | 19.51 | <1.0$\times{10}^{-50}$ |
| $\alpha$ | 3.364171e-01 | 1.764275e-02 | 3.018e-01,3.710e-01) | 19.07 | <1.0$\times{10}^{-50}$ |
| $\lambda$ | 1.163292e+00 | 3.018496e-03 | 1.157e+00, 1.169e+00) | 385.39 | <1.0$\times{10}^{-50}$ |
| $x_{b}$ | 4.474265e+01 | 1.227057e+00 | (42.337, 47.148) | 36.46 | <1.0$\times{10}^{-50}$ |
| $p$ | 1.405237e-01 | 5.817952e-03 | (1.291e-01, 1.519e-01) | 24.15 | <1.0$\times{10}^{-50}$ |
| $\sigma_{1}$ | 1.242761e+00 | 2.927e-02 | (1.18543, 1.30017) | 42.46 | <1.0$\times{10}^{-50}$ |
| $\sigma_{2}$ | 2.703559e-01 | 2.783e-03 | (2.649e-01, 2.758e-01) | 97.15 | <1.0$\times{10}^{-50}$ |
| logLikMx | 50898.9 |  |  |  |  |
| AIC | -101783.8 |  |  |  |  |

### Fitting statistics for a (EMEM, $\boldsymbol{w}_{\boldsymbol{BH}},\boldsymbol{h}_{\boldsymbol{p}}\left( \boldsymbol{x},\boldsymbol{c} \right),\boldsymbol{NM}_{\boldsymbol{2}})$ protocol

In order to arrange a (EMEM, $w_{BH},1, N)$ scheme in the settings of the EMEM of Equation (4) we let $w(x,p)$ holding a biphasic form, namely, $w\left( x,p \right)=w_{BH}(x,p)$ (c.f. Equation (6)), we also maintain a $\epsilon\sim{NM}_{2}\left( 0,s \right)$ assumption, but the variance scaling function $h\left( x,c \right)$ switches to being expressed in a piecewise form $h_{p}\left( x,c \right)$, (c.f. Equation (9)), namely,

$h_{p}\left( x,c \right)=\left\{ \begin{matrix} 1, if, 0<x\leq x_{b} \\ \left( \frac{x_{b}}{x} \right)^{c}, if, x_{b}<x \end{matrix} \right.$ (9)

where $x_{b}$ is the breakpoint associating to $w_{BH}\left( x,y \right)$ (c.f. Equation (6)). Table 8 gives the associating fitting statistics.

**Table 8**. **Fitting statistics associating to a (EMEM,** $\boldsymbol{w}_{\boldsymbol{BH}}\boldsymbol{,}\boldsymbol{h}_{\boldsymbol{p}}\left( \boldsymbol{x}\boldsymbol{,}\boldsymbol{c} \right)\boldsymbol{,}\boldsymbol{NM}_{\boldsymbol{2}}$**) scheme**. In this table we include parameter estimates with their projected uncertainties, t-value, p-value, LogLikMx and AIC stand for Maximum Loglikelihood and Akaike information index values one to one.

| **Parameter** | **Estimate** | **Std. Err.** | **Confidence Interval (95%)** | **t-value** | **p-value** |
| --- | --- | --- | --- | --- | --- |
| $\beta$ | 0.000129314 | 8.5640e-06 | (1.1253e-04, 1.4610e-04) | 15.10 | $<2\times{10}^{-15}$ |
| $\alpha$ | 0.287398329 | 2.4222e-02 | (2.3992e-01, 3.3487e-01) | 11.87 | $<2\times{10}^{-15}$ |
| $\lambda$ | 1.158905602 | 2.7136e-03 | (1.1536e+00, 1.1642e+00) | 427.08 | $<2\times{10}^{-15}$ |
| $x_{b}$ | 36.40843191 | 1.2900e+00 | (3.3880e+01, 3.8937e+01) | 28.22 | $<2\times{10}^{-15}$ |
| $p$ | 0.128403772 | 1.6169e-02 | (9.6712e-02, 1.6010e-01) | 7.94 | $<2\times{10}^{-15}$ |
| $\sigma_{1}$ | 1.727801581 | 5.1227e-02 | (1.6274e+00, 1.8282e+00) | 33.73 | $<2\times{10}^{-15}$ |
| $\sigma_{2}$ | 0.50277001 | 1.0268e-02 | (4.8264e-01, 5.2289e-01) | 48.96 | $<2\times{10}^{-15}$ |
| $c$ | 0.2665986 | 6.8834e-03 | (2.5311e-01, 2.8009e-01) | 38.73 | $<2\times{10}^{-15}$ |
| logLikMx | 51610.8 |  |  |  |  |
| AIC | -103205.6 |  |  |  |  |

### Assessment of Reproducibility Strength of Considered Allometric Proxies

For comparison aims, in Table 9 bellow we provide statistics for the valuation of the reproducibility power of EMEM derived allometric proxies for observed monthly average leaf biomass in shots reported in the present data. In Table 9 we make available AIC, Standard Error of Estimate (SEE), and Mean Prediction Error (MPE) [32-35], see the main text for references). Similarly, we bring in Lin’s Concordance Correlation Coefficient, denoted here through the CCCsymbol [31-36], see the main text for references). We propose a Relative Absolute Deviation index value (RAD) [29]. To derive RAD statistics, we begin by finding the difference between the average monthly leaf biomass values obtained from the allometric method and the overall average of the observed leaf biomass values. Then, we divide this difference by the overall average of the observed individual leaf biomass values. Calculation of reproducibility measures for the ELM based methods relied in retransformation of ensuing protocols fitted in geometrical space.

**Table 9**. **Assessment of reproducibility strength of EMEM based allometric projection methods**. We provide AIC, CCC, SEE, MPE, MPSE and RD statistics.

| **Model** | **Table** | **AIC** | **CCC** | **SEE** | **MPE** | **MPSE** | **RD** |
| --- | --- | --- | --- | --- | --- | --- | --- |
| ($EM,w_{H},1,N$) | 1 | -94745.76 | 0.9174 | 0.0039 | 15.02 | 16.74 | 0.1410 |
| (EM, $w_{BH}$ $,1, N)$ | 2 | -96385.61 | 0.9860 | 0.0018 | 7.0620 | 11.0505 | 0.0017 |
| (EM, $w_{BH},1,{NM}_{2})$ | 3 | -101783.8 | 0.9733 | 0.0026 | 10.1060 | 13.0626 | 0.0024 |
| (EM, $w_{BH},h_{p},{NM}_{2})$ | 4 | -103205.6 | 0.9890 | 0.0015 | 5.8609 | 7.3163 | 0.0013 |

Statistics included in Table 9 corroborate the outmost reproducibility strength offered by the (EM, $w_{BH},h_{p},{NM}_{2})$scheme.
